# In Search of Factors Determining Activity of Co_3_O_4_ Nanoparticles Dispersed in Partially Exfoliated Montmorillonite Structure

**DOI:** 10.3390/molecules26113288

**Published:** 2021-05-29

**Authors:** Anna Rokicińska, Tomasz Berniak, Marek Drozdek, Piotr Kuśtrowski

**Affiliations:** Faculty of Chemistry, Jagiellonian University, Gronostajowa 2, 30-387 Kraków, Poland; anna.rokicinska@uj.edu.pl (A.R.); tomasz.berniak@doctoral.uj.edu.pl (T.B.); marek.drozdek@uj.edu.pl (M.D.)

**Keywords:** catalytic total oxidation, toluene, Co_3_O_4_, montmorillonite, hydrogel-assisted modification

## Abstract

The paper discusses a formation of Mt–PAA composite containing a natural montmorillonite structure partially exfoliated by poly(acrylic acid) introduced through intercalation polymerization of acrylic acid. Mt–PAA was subsequently modified by controlled adsorption of Co^2+^ ions. The presence of aluminosilicate packets (clay) and carboxyl groups (hydrogel) led to the deposition of significant amounts of Co^2+^ ions, which after calcination formed the Co_3_O_4_ spinel particles. The conditions of the Co^2+^ ions’ deposition (pH, volume and concentration of Co(NO_3_)_2_ solution, as well as a type of pH-controlling agent) were widely varied. Physicochemical characterization of the prepared materials (including X-ray fluorescence (XRF), X-ray powder diffraction (XRD), low-temperature nitrogen adsorption, X-ray photoelectron spectroscopy (XPS) and temperature-programmed reduction (H_2_-TPR)) revealed that the modification conditions strongly influenced the content as well as the distribution of the Co_3_O_4_ active phase, tuning its reducibility. The latter parameter was, in turn, very important from the point of view of catalytic activity in the combustion of aromatic volatile organic compounds (VOCs) following the Mars–van Krevelen mechanism.

## 1. Introduction

The rapid development of technology has brought about growing concentrations of volatile organic compounds (VOCs) in the atmosphere. The organic pollutants are emitted from various natural (e.g., decomposition of biomass) and anthropogenic sources (e.g., oil refineries, fuel combustion, chemical industry, painting, printing and solvent processing, as well as production of pharmaceutics, automobiles, clothes, coatings or furniture). In the total emission of VOCs, different aromatic compounds, such as benzene, toluene, xylenes or ethylbenzene, play an important role [1,2]. Due to their toxic and harmful effects, strict regulations related to a permissible emission level have been implemented. Moreover, released VOCs can be eliminated by many developed physical, chemical and biological methods. However, apart from the adsorption processes, the best results are usually achieved using thermal oxidation, catalytic oxidation or photocatalytic degradation [3,4].

The catalytic oxidation offers several advantages compared to the thermal oxidation, especially at low VOC concentrations. This approach enables the complete destruction of VOCs at significantly lower temperatures (250–500 °C) without the formation of undesirable by-products [1,5]. Nevertheless, adequately active, selective and stable catalysts are required. Extremely active noble metal-containing materials (e.g., Pt [6,7], Pd [8,9] or Au [10]) are not an ideal choice due to their poisoning tendency and high cost of production. Therefore, alternative transition metal oxide-based catalysts have been intensively investigated in the combustion of VOCs [11]. Among them, materials containing the Co_3_O_4_ spinel phase were recognized as one of the most promising catalysts. It is not surprising that various forms of this spinel were studied, including, for example, nanometric one- (nanodots), two- (nanosheets) and three-dimensional systems [12,13,14,15,16,17]. The aim is to expose the surface of the formed material as much as possible, hence using the leaf-templated method for the formation of 3D hierarchical porous Co_3_O_4_ proposed by Han et al. [18] seemed very efficient. The irregularly shaped Co_3_O_4_ nanoparticles were combined to ultimately form a multi-layered, porous 3D structure with a highly specific surface area and availability of catalytically active sites. A similar effect was achieved using the nanocasting route with hard templates (such as KIT-6 or SBA-15) [19,20]. Not only that, the use of an ordered, mesoporous silica template provided a defined pore geometry and diameter in the formed Co_3_O_4_ replicas. The porous structure of Co_3_O_4_ facilitates diffusion of reagent molecules and their adsorption on active centers, the number of which is appropriately high due to the developed specific surface.

Unfortunately, a significant disadvantage of pure Co_3_O_4_ catalysts for the VOCs’ combustion is a sintering effect observed during the reaction. Hence, active oxide particles are very often deposited on various supports with appropriate thermal and mechanical stability as well as a developed surface. The supports most frequently used for the deposition of Co_3_O_4_ are based on different individual metal oxides (e.g., Al_2_O_3_ [21,22,23], SiO_2_ [24], TiO_2_ [25,26,27], CeO_2_ [28,29], ZrO_2_ [30], Fe_2_O_3_ [31]), nitrogen-doped carbons [32,33,34], zeolites [35,36], perovskites [37] or hydrotalcites [38,39].

Important parameters that influence the activity of Co_3_O_4_-based catalysts include the amount and morphology of this spinel phase [40]. Wang et al. [41] found that the catalytic performance strongly depended on dimensional architectures of Co_3_O_4_ synthesized in the form of octahedrons, plates and rods. The Co_3_O_4_ rods catalyst with primarily exposed (110) facets exhibited the best catalytic activity in the CO oxidation because there were more oxygen defects on its surface. Similar conclusions were given by Ma and co-workers [42]. Mesoporous Co_3_O_4_ with the most exposed (110) facets displayed higher catalytic activity in the ethylene oxidation compared to Co_3_O_4_ nanosheets with the dominance of (112) facets. It was found that the (110) planes composed mainly of Co^3+^ cations provided sufficient sites for ethylene and oxygen adsorption. The appropriate exposure of the Co_3_O_4_ facets also influenced the catalytic activity in the toluene combustion, shifting the temperature window of the reaction even by ~20 °C [43]. From the point of view of catalytic activity in the VOCs’ combustion, surface concentration of O_ads_ groups and porosity of a catalyst, as well as its proper reducibility, are also important. The appearance of O_ads_ is related to the generation of oxygen vacancies resulting from the defective Co_3_O_4_ structure. The defects distributed in the spinel structure facilitate adsorption of O_2_ and the formation of active oxygen species [44]. Thus, many authors claimed that the presence of Co_3_O_4_ spinel with a large amount of easily reducible Co^3+^ cations is essential to obtain high catalytic activity in the total oxidation of VOCs [43]. Generally, Co^3+^ ions can act as active sites for toluene combustion and influence the charge imbalance detected in O 1s XPS spectra [44].

The catalytic combustion of VOCs on Co_3_O_4_ catalysts is usually described by the Mars–van Krevelen mechanism, in which lattice oxygen plays a significant role. The factors that limit this mechanism are the number of reactive oxygen species, the number of active sites and the type of metal ions at high oxidation states (such as Co^3+^) [32]. In situ FT-IR studies allowed researchers to postulate the plausible mechanism of benzene oxidation on Co_3_O_4_/eggshell catalysts [45]. In the first step, benzene molecules are adsorbed on cobalt active centers located on the catalyst surface, resulting in the formation of phenolic compounds, which is enhanced by the presence of the active O_latt_ and O_ads_ species. The phenolic compounds are characterized by high reactivity due to the presence of hydroxyl functional groups easily forming benzoquinones. In a subsequent step, the ring opens to form acetate and maleate forms, which are further deeply oxidized to CO_2_ and H_2_O. Furthermore, Wang and co-authors [24] investigated the geometrical-site-dependent catalytic activity of Co^3+^ and Co^2+^ by substituting these metal ions with inactive or less active Zn^2+^ (d^0^), Al^3+^ (d^0^) and Fe^3+^ (d^5^) cations. The collected results demonstrated that octahedrally coordinated Co^2+^ sites are more easily oxidized to Co^3+^ species compared to tetrahedral Co^2+^. It was found that Co^3+^ sites are responsible for the oxidative breakage of benzene rings towards carboxylate intermediate species.

Zhong et al. [46] studied the mechanism of toluene combustion by in situ DRIFTS combined with PTR-TOF-MS. They claimed that toluene molecules adsorbed on the Co_3_O_4_ surface react with lattice oxygen to form benzyl alcohol, which is subsequently oxidized to benzaldehyde, benzoic acid and benzene. The presence of active oxygen favors further oxidation to phenol and benzoquinone. After ring opening in benzoquinone, maleic anhydride species are formed. All mentioned organic species can be ultimately oxidized to CO_2_ and H_2_O. Co^2+^ ions which appear on the surface due to reduction are re-oxidized to Co^3+^ by oxygen molecules present in the gas phase. The replenished O_latt_ is able to participate in the next reaction cycle.

In our previous work [47] we signaled that very efficient Co_3_O_4_-based catalysts for the elimination of aromatic VOCs can be obtained using natural layered aluminosilicates (specifically montmorillonite) partially exfoliated by intercalation of a hydrogel network based on poly(acrylic acid) (Mt–PAA). The polymer–clay composites constructed in this way can be easily modified with metal cations due to the presence of negatively charged montmorillonite layers and carboxyl groups in the polymer chains. The adsorption conditions strongly determine the amount and distribution of the introduced cations. In the mentioned paper we disclosed an influence of pH on adsorption of Co^2+^ from a cobalt nitrate solution used as an active phase precursor, and the catalytic activity of the corresponding materials after calcination. The best catalytic results were achieved for the catalysts based on Mt–PAA modified within the pH range of 5.0 to 8.0. The Co^2+^ adsorption at lower pH is poorly efficient mainly due to the presence of a limited number of dissociated COO^-^ species in the hydrogel part of the composite. On the other hand, the increase in pH above 8.0 favors uncontrolled precipitation of Co^2+^ hydroxide, which upon thermal treatment is subsequently decomposed to Co_3_O_4_ particles characterized by low catalytic activity in the toluene combustion. Nevertheless, other synthesis parameters may also be crucial for the catalytic performance. Hence, in the present work, we have decided to show the results of a study on the effect of concentration and amount of the introduced modifier solution, as well as a chemical agent controlling pH in the range of 5.0 to 8.0 used for the modification of Mt–PAA composite containing equal amounts of clay (50 wt%) and hydrogel (50 wt%). The collected conclusions seem very important for the further development of catalysts dedicated to the aromatic VOCs’ combustion.

## 2. Results and Discussion

### 2.1. Effect of Volume of Co^2+^ Solution

As mentioned above, the deeper investigation on the effects influencing the catalytic performance of Co^2+^-modified Mt–PAA composite-derived materials was limited only to the samples produced under optimal pH conditions (5.0–8.0). Consequently, different amounts of Co^2+^ cations were introduced by a contact of the Mt–PAA composite with various volumes (100–700 mL) of 0.01 M solution of Co^2+^ nitrate using NaOH as the pH-controlling agent.

**Figure 1:**
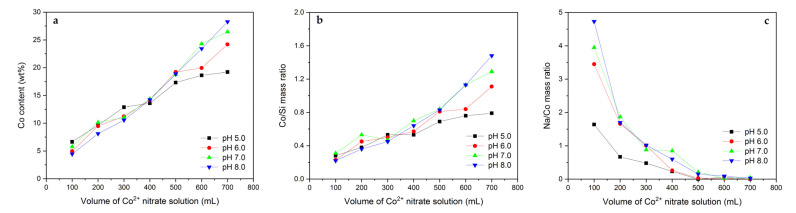
(**a**) Co content as well as (**b**) Co/Si and (**c**) Na/Co mass ratios in the calcined Mt–PAA composite modified in various volumes of 0.01 M Co(NO_3_)_2_ solution using NaOH as the pH-controlling agent.

The real Co contents in the calcined materials are compared in Figure 1a. It is obvious that the volume of Co^2+^ nitrate solution used during the adsorption influenced the Co loading. The observed relationships are fairly linear, and the highest Co contents (26.4 and 28.3 wt%) were achieved after the deposition in 700 mL of the Co(NO_3_)_2_ solution kept at a constant pH equaling 7.0 and 8.0, respectively. On the other hand, these tendencies should be reflected in the measured Co/Si mass ratios (Figure 1b). The collected data generally meet this expectation with the exception of the samples modified at the highest pH. The materials treated at pH = 8.0 exhibit a noticeable increase in the Co/Si mass ratio, which suggests a partial desilication of the Mt support. Furthermore, using NaOH as the pH-controlling agent led to an appearance of Na in the calcined samples. The content of Na in relation to the Co loading is analyzed in Figure 1c. The raising pH results in higher Na/Co mass ratios due to the introduction of higher amounts of NaOH in order to attain the intended OH^-^ concentration. Additionally, a decrease in the Na/Co mass ratios with volume of the Co(NO_3_)_2_ solution is observed. This effect can be explained by the chemical composition of solution containing the Mt–PAA composite after the adjustment to the proper pH. Depending on the modification conditions and volume of the Co(NO_3_)_2_ solution, different amounts of NaOH were added, resulting in varying ratios of Na^+^/Co^2+^ cations. At lower volumes of the Co(NO_3_)_2_ solution, concentration of Na^+^ ions remained in excess, reflecting the higher Na/Co mass ratios in the final product. Decreasing pH led to the decline in Na content in the modifying solution and consequently in its lower loading in the calcined samples. Nevertheless, the observed relations undoubtedly confirm that the deposition of Co^2+^ ions competes with the Na^+^ adsorption and begins to play a significant role under appropriately chosen modification conditions (low pH, high volume of Co(NO_3_)_2_ solution).

**Figure 2:**
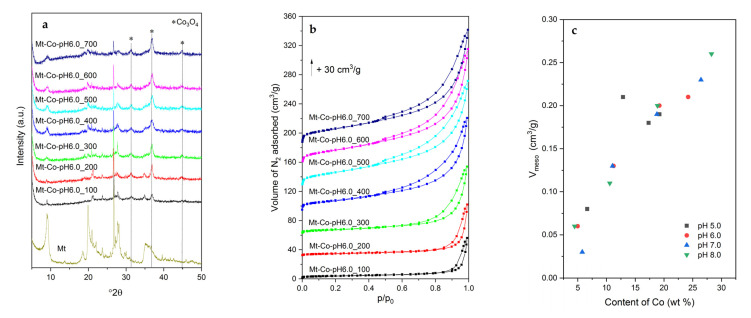
(**a**) XRD patterns, (**b**) N_2_ adsorption–desorption isotherms and (**c**) correlation between mesopore volume and Co content for the calcined Mt-Co-pH6.0 samples obtained by deposition in various volumes of 0.01 M Co(NO_3_)_2_ solution at pH = 6.0 using NaOH as the pH-controlling agent.

The structure of the calcined materials was studied by XRD. Examples of XRD patterns collected for the catalysts based on the MT–PAA composite modified at pH = 6.0 are shown in Figure 2a. The diffraction pattern of dried montmorillonite is additionally presented.

After the thermal treatment, the (001) diffraction peak, typical of the layered structure of Mt, is found at 9.6 °2θ. However, a considerable reduction in its intensity is observed, which can be explained by dilution of the clay with a Co-containing phase, fluorescence effect related to the presence of Co in the measured samples and, what is most important, partial delamination of the montmorillonite structure by previous intercalation with PAA followed by adsorption of Co^2+^ cations. For the Co-modified materials, three diffraction peaks at 31.4, 36.9 and 44.9 °2θ are detected, corresponding to the regular structure of Co_3_O_4_ spinel (PDF 00-009-0418). The XRD measurements did not unveil an existence of any further crystalline phases containing Co.

An interesting insight into the textural properties of the studied catalysts is given by the low-temperature N_2_ adsorption measurements. In Figure 2b, some examples of adsorption–desorption isotherms for the series of Mt-Co-pH6.0 samples are demonstrated. For the materials prepared with relatively low volumes of Co^2+^ nitrate solution, which contain small amounts of the spinel phase, the shape of the recorded isotherms reveals their poorly porous nature with a dominant role of interparticle voids (adsorption of N_2_ at the highest relative pressures *p*/*p*_0_). Increasing the Co content brings about the enhancement of N_2_ adsorption at lower *p*/*p*_0_, attributed to the formation of mesopores in the structure of partially delaminated Mt (in these cases the isotherms are clearly of type IV with the H3 type of hysteresis loop). It should be therefore supposed that a higher number of Co_3_O_4_ nanoparticles was intercalated between the Mt layers, resulting in the so-called house of cards structure. One can expect that during deposition of Co^2+^ cations, water present in the system plays a double role: (i) solvent of nitrate salt, and (ii) swelling agent. When a low volume of H_2_O is contacted with the Mt–PAA composite, the structure of the hydrogel is insufficiently penetrated by the swelling agent, and the deposition of Co^2+^ occurs mainly close to the external surface of the Mt–PAA particles. Raising the Co(NO_3_)_2_ solution volume results in enhanced swelling of the hydrogel and all parts of the Mt–PAA particles become enriched in Co^2+^ ions. These differences are reflected by the distribution and size of formed Co_3_O_4_ nanoparticles. They are relatively large and occluded mainly in the voids between the Mt grains (the samples modified at low Co(NO_3_)_2_ solution volume). Better penetration of the Mt–PAA composite with Co^2+^ cations brings about higher dispersion of spinel nanoparticles, which also appear in the pores formed by partial exfoliation of the layered structure of montmorillonite. This picture is clearly confirmed by the changes in textural parameters of the Co-modified materials (Table 1). The developed samples show a limited number of micropores with a volume below 0.01 cm^3^/g. Significantly higher volumes of mesopores are observed, which additionally increase with raising the Co content (Figure 2c). The values of V_meso_ above 0.20 cm^3^/g are achieved for the samples modified at the highest volumes of 0.01 M Co(NO_3_)_2_ solution. The presence of mesopores results in expanded surface area, which for the materials with optimal mesoporosity is close to 100 m^2^/g.

**Figure 3:**
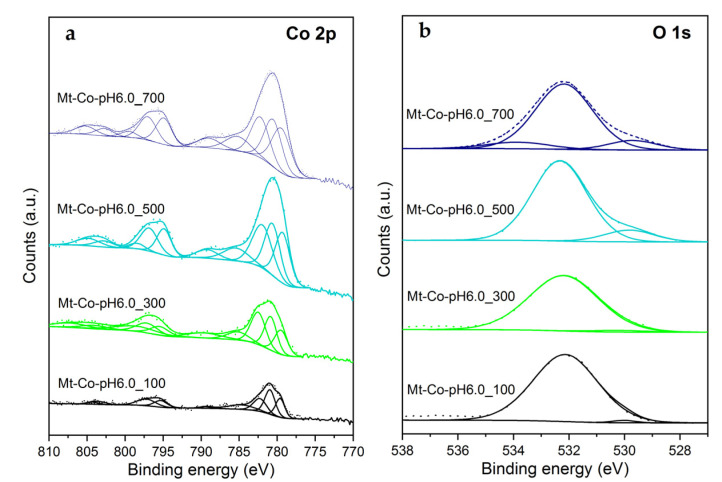
XPS Co 2p (**a**) and O 1s (**b**) spectra of the calcined Mt-Co-pH6.0 samples obtained by deposition in various volumes of 0.01 M Co(NO_3_)_2_ solution at pH = 6.0 using NaOH as the pH-controlling agent.

The surface composition of the developed materials was investigated by XPS. The chosen XPS Co 2p and O 1s spectra for the Mt-Co-pH6.0 samples are demonstrated in Figure 3.

Furthermore, the contents of elements determined on the surface of all materials by analysis of corresponding regions (including Na 1s, Co 2p, Si 2p and Al 2p) are comparatively presented in Table 2.

In the Co 2p spectra, the characteristic doublet of Co 2p_3/2_ and Co 2p_1/2_ peaks with the spin-orbital splitting of 15.2–15.6 eV. Nevertheless, the components corresponding to Co^2+^ in tetrahedral coordination (at 780.8 ± 0.3 eV for Co 2p_3/2_) and Co^3+^ in octahedral coordination (at 779.5 ± 0.3 eV for Co 2p_3/2_), typical of Co_3_O_4_, are distinguished [48]. Additionally, due to the presence of Co^2+^ ions, a third component related to multiplet splitting is identified at 782.2 ± 0.3 eV [49].

The XPS results confirm the conclusions from chemical analysis by XRF. The increase in the Co(NO_3_)_2_ solution volume used during modification brings about raising the Co loading at a reduction in Na amounts. These changes are accompanied by an increase in participation of the O 1s component at 530.0 ± 0.3 eV, attributed to O^2-^ lattice oxygen, in relation to another component at 532.2 ± 0.3 eV assigned to surface hydroxyls. The Co^2+^/Co^3+^ molar ratio varies within a relatively wide range of 1.0 to 2.3 for the studied catalysts. The determined values still fit well to the ratios typical of Co_3_O_4_, which have been previously presented in the literature [50,51]. However, no straight correlation between the Co^2+^/Co^3+^ molar ratios and the synthesis conditions is observed. The comparison of the Co/Si molar ratios in the materials’ bulk (determined by XRF) and on their surface (determined by XPS) is especially worth stressing (Table 2). A vast majority of the studied materials shows the higher bulk Co content compared to the surface one. Nevertheless, these differences are more distinct for the materials prepared using the larger volumes of the Co(NO_3_)_2_ solution. Obviously, in the case of these samples more highly dispersed Co_3_O_4_ nanoparticles were introduced into the clay structure, providing its delamination and development of porosity, confirmed by XRD and N_2_ adsorption.

**Figure 4:**
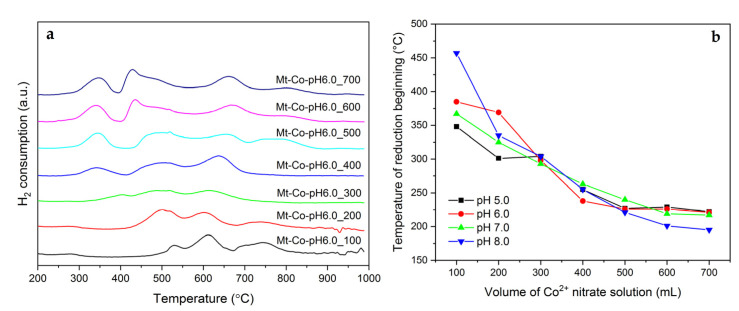
(**a**) H_2_-TPR profiles of the calcined Mt-Co-pH6.0 samples obtained by deposition in various volumes of 0.01 M Co(NO_3_)_2_ solution using NaOH as the pH-controlling agent, and (**b**) corresponding temperatures of reduction beginning determined from the H_2_-TPR measurements.

The calcined samples synthesized by deposition of Co^2+^ cations in the different volumes of the Co(NO_3_)_2_ solution were also studied by H_2_-TPR. The recorded H_2_-TPR profiles for the Mt-Co-pH6.0 materials are shown in Figure 4a.

The reduction of Co_3_O_4_ is usually very sensitive to its dispersion and the strength of interaction with a support [52]. Typically, the first step of this process is attributed to the reduction of Co^3+^ to Co^2+^, forming CoO, which can be strongly stabilized on the support surface [53,54]. The subsequent deep reduction to metallic Co is undergone at higher temperatures. The stabilization of the spinel phase is also clearly observed in the case of the studied catalysts. The reduction of Co_3_O_4_ is found below 550 °C, when the clay dehydroxylation occurs. However, the temperature of Co_3_O_4_ reduction beginning significantly depends on the conditions of modification of Mt–PAA with Co^2+^ cations (Figure 4b). For the materials obtained by deposition in low volumes of 0.01 M Co(NO_3_)_2_ solution, the occlusion of Co_3_O_4_ nanoparticles between the Mt grains and its low content cause very strong stabilization of the spinel phase, and its reduction requires temperatures above 350 °C. The highest stabilization is detected for the Mt-Co-pH8.0_100 material, which contains Co_3_O_4_ reduced even above 450 °C. On the other hand, the samples modified in 600 and 700 mL of 0.01 M Co(NO_3_)_2_ solution with the highest dispersion of Co_3_O_4_ nanoparticles and Co loading show the lowest reduction temperatures. It should be moreover noticed that apart from the easily reducible spinel phase, in the synthesized samples there are other Co-containing species, which are reduced at significantly higher temperatures (above 550 °C). In the case of these forms, an opposite tendency in proneness to reduction, in relation to the above-discussed effects of Co_3_O_4_ reduction, is observed. A higher Co concentration in the studied material leads to stronger stabilization of Co-containing species during reduction above 550 °C. The presence of these species is not surprising due to a possibility of direct interaction of introduced Co^2+^ cations with the clay by electrostatic attraction, their incorporation into the Mt sheets and/or a formation of extra framework mixed oxides.

**Figure 5:**
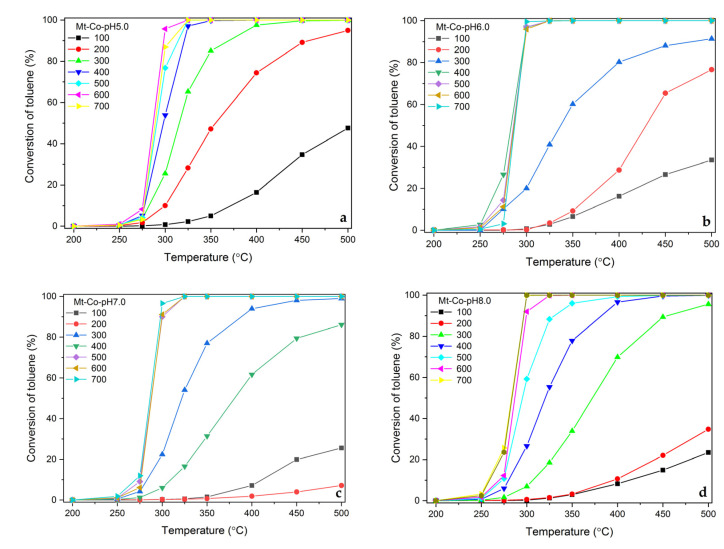
Influence of reaction temperature on toluene conversion over the calcined Mt-Co-pH5.0 (**a**), Mt-Co-pH6.0 (**b**), Mt-Co-pH7.0 (**c**) and Mt-Co-pH8.0 (**d**) samples obtained by deposition in various volumes of 0.01 M Co(NO_3_)_2_ solution using NaOH as the pH-controlling agent.

Finally, the catalytic performance of the synthesized Co-doped materials in the total oxidation of toluene was studied. In addition to high activity in the tested reaction, the catalysts showed a very high selectivity to the desired products, i.e., CO_2_ and H_2_O. The materials prepared by adsorption of Co^2+^ ions in the pH range from 5.0 to 8.0 make it possible to keep the selectivity to benzene below 0.1%. Furthermore, over no catalyst the formation of CO was detected under the reaction conditions used. Figure 5 demonstrates the collected results in the form of toluene conversion expressed as a function of increasing reaction temperature.

**Figure 6:**
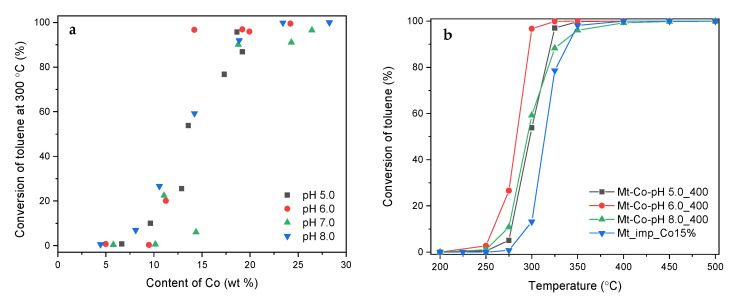
(**a**) Influence of Co content on conversion of toluene achieved at 300 °C over the Mt-Co-pHx catalysts obtained by deposition in 0.01 M Co(NO_3_)_2_ solution of various volumes using NaOH as the pH-controlling agent, and (**b**) comparison of catalytic activity for Mt-Co-pHx containing ca. 15 wt% of Co with Mt impregnated with the same Co loading (Mt_imp_Co15%).

As expected, the catalytic activity increases with rising Co content, achieved by deposition in higher volumes of the Co(NO_3_)_2_ solution. This results in the formation of larger amounts of Co_3_O_4_ phase nanocrystallites exhibiting easy reducibility desirable from the point of view of the reaction occurring according to the Mars–van Krevelen mechanism. The effect of the Co content on the catalytic activity (measured by the conversion of toluene at 300 °C) is clearly shown in Figure 6a.

The catalysts containing less than 10 wt% of Co give rather low toluene conversion. On the other hand, the materials with the Co contents exceeding 18 wt% allow us to achieve a toluene conversion of over 85% under these reaction conditions. Therefore, the catalysts with the medium Co loadings have a great potential for further research. In this case, an appropriate preparation method may be a key parameter determining the performance in the aromatic VOCs’ combustion. Figure 6b compares the catalytic activity of the developed Mt-Co-pHx materials containing approx. 15 wt% of Co with natural montmorillonite modified by dry impregnation using an aqueous solution of Co(NO_3_)_2_ in an amount allowing the introduction of the same active phase content (Mt_imp_Co15%). As can be seen from the presented relationships, the catalysts prepared by the hydrogel-assisted route exhibit considerably higher activity compared to Mt_imp_Co15%. The best results were noted for the material obtained by adsorption of Co^2+^ ions carried out in 400 mL of the Co(NO_3_)_2_ solution at controlled pH = 6.0. Its high activity is strongly correlated with enhanced reducibility of the formed Co_3_O_4_ phase (cf. Figure 4b).

**Figure 7:**
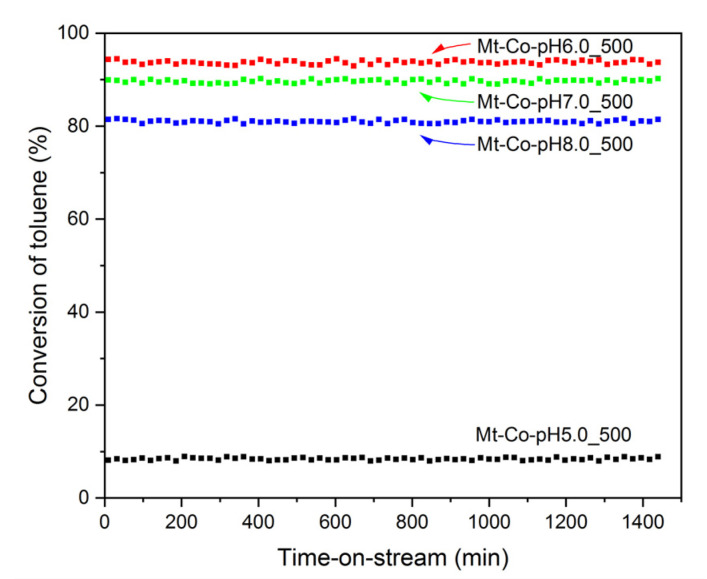
Stability of the chosen Mt-Co-pHx_500 catalysts obtained by deposition in 0.01 M Co(NO_3_)_2_ solution using NaOH as the pH-controlling agent during the isothermal tests in the toluene combustion performed at 290 °C.

Stability tests were also carried out in the toluene combustion at 290 °C for 24 h. Figure 7 illustrates the collected results in the form of toluene conversion over the catalysts obtained from the precursors modified in 500 mL of 0.01 M Co(NO_3_)_2_ solution at different pH (5.0, 6.0, 7.0 and 8.0). The high stability of the tested materials is noteworthy. The initial toluene conversions of 8.4, 93.7, 89.6 and 81.0% for Mt-Co-pH5.0_500, Mt-Co-pH6.0_500, Mt-Co-pH7.0_500 and Mt-Co-pH8.0_500, respectively, remain almost unchanged during the whole test. The observed high stability of the tested catalysts should be attributed to the presence of delaminated montmorillonite packages, which, evenly distributed throughout the entire volume of the material, protect against aggregation of Co_3_O_4_ nanoparticles, maintaining a high degree of their dispersion. To conclude, the high activity and selectivity combined with no deactivation make the developed materials very useful for commercial technologies dedicated to the elimination of aromatic VOCs.

### 2.2. Effect of Concentration of Co^2+^ Solution

**Figure 8:**
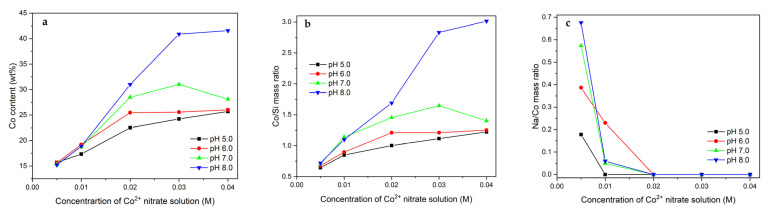
(**a**) Co content as well as (**b**) Co/Si and (**c**) Na/Co mass ratios in the calcined Mt–PAA composite modified in 500 mL of Co(NO_3_)_2_ solution with different concentrations using NaOH as the pH-controlling agent.

The analysis of optimal parameters for modification of the Mt–PAA composite with Co^2+^ ions was extended by the concentration of the Co(NO_3_)_2_ solution, changed in the range from 0.005 to 0.040 M. Adsorption was performed maintaining a constant volume of the modifying solution (500 mL) at four different pH (5.0–8.0). The chemical composition of the materials obtained after calcination at 500 °C was checked by XRF. Figure 8 shows the determined Co content as well as the Co/Si and Na/Co mass ratios. As can be seen, the concentration of the Co(NO_3_)_2_ solution has a very significant effect on the amount of deposited Co. In the case of the solution with the lowest concentration (0.005 M), regardless of pH of the solution, similar Co contents are found in the final catalysts (15.2–15.7 wt%). The increase in the concentration of the Co(NO_3_)_2_ solution results in a gradual increase in the Co loading. After deposition in the solution with the highest concentration (0.040 M) at lower pH, the Co contents of 25.7–28.1 wt% are attained. For the highest pH, this value is much higher and even reaches 41.6% by weight.

In addition to cobalt, noticeable amounts of sodium are also introduced during the modification. Figure 8c presents the Na/Co mass ratio for a series of synthesized materials. The highest concentrations of Na are noted for the materials treated in the Co(NO_3_)_2_ solutions with the low concentrations (especially 0.005 M) at the highest pH. This fact can be explained, as in the case of the discussion related to the Na content in the preparations modified in the 0.01 M Co(NO_3_)_2_ solution of different volumes, by the competitive adsorption of Na^+^ and Co^2+^ ions. In the solution where Na^+^ ions play a dominant role, they are incorporated to a greater extent into the structure of the Mt–PAA composite. As the concentration of the Co(NO_3_)_2_ solution increases, the Na^+^/Co^2+^ ratio in the modifying solution decreases, and therefore for the Mt-Co-pHx_500_0.020, Mt-Co-pHx_500_0.030 and Mt-Co-pHx_500_0.040 materials, no significant loadings of Na are observed.

**Figure 9:**
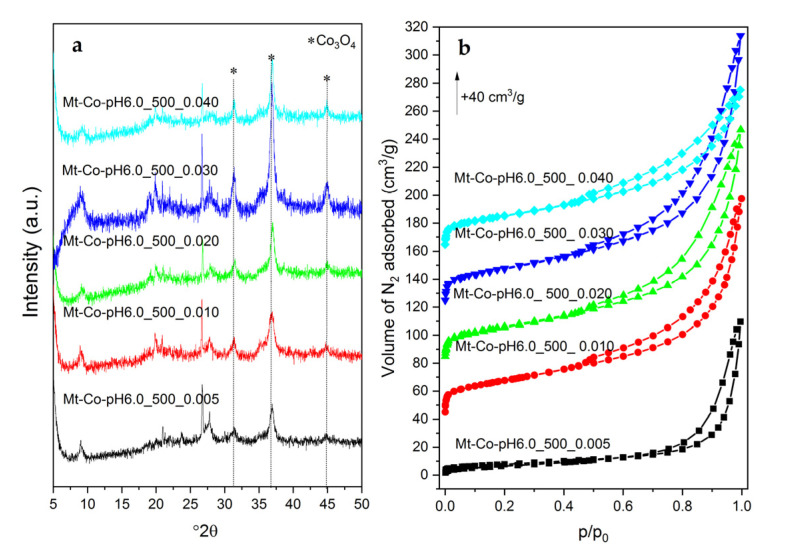
XRD patterns (**a**) and N_2_ adsorption–desorption isotherms (**b**) of the calcined Mt-Co-pH6.0_500 samples obtained by deposition in various concentrations of Co(NO_3_)_2_ solution (500 mL) at pH = 6.0 using NaOH as the pH-controlling agent.

The change in the concentration of the modifying solution did not significantly affect the crystalline structure of the obtained materials (see example diffraction patterns collected for the Mt-Co-pH6.0_500 catalysts in Figure 9a). Regardless of the concentration of the Co(NO_3_)_2_ solution and pH, partial delamination of the layered clay structure due to the appearance of spinel phase nanoparticles is observed. The reflections attributed to the Co_3_O_4_ structure in the case of the materials modified in the solutions with higher Co(NO_3_)_2_ concentrations become more intense despite the fluorescence effect. This may indicate a greater Co aggregation in the formed spinel species, which is reasonable due to increasing Co content with rising concentration of the modifying solution and more compact distribution of adsorbed Co^2+^ ions in the PAA network.

Low-temperature nitrogen adsorption was used to determine the porosity of the synthesized materials. The collected isotherms for the chosen Mt-Co-pH6.0_500 catalysts are given in Figure 9b. An increase in the amount of N_2_ adsorbed on the surface of the measured samples in the range of average relative pressures (*p*/*p*_0_) along with the increase in the concentration of the modifying solution from 0.005 to 0.010–0.030 M indicates the opening of the porous structure as a result of the formation of a larger number of Co_3_O_4_ nanoparticles. However, it is worth noting that too high concentration of the Co(NO_3_)_2_ solution is not favorable from the point of view of forming porosity. Most likely, too-large spinel phase crystallites present in the structure of the calcined Mt–PAA composite modified with cobalt phase fill interparticle voids, reducing the pore volume and specific surface area (Table 3).

**Figure 10:**
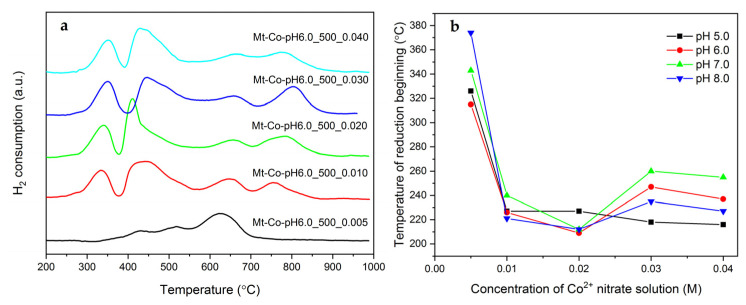
(**a**) H_2_-TPR profiles of the calcined Mt-Co-pH6.0_500 samples obtained by deposition in various concentrations of Co(NO_3_)_2_ solution using NaOH as the pH-controlling agent, and (**b**) corresponding temperatures of reduction beginning determined from the H_2_-TPR measurements.

The DTG curves of the Mt-Co-pH6.0_500 samples collected at reducing atmospheres of Ar/H_2_ (Figure 10a) show the presence of the typical, earlier-discussed effects of weight loss, which correspond to: (i) gradual reduction of Co_3_O_4_ to Co^0^ (210–550 °C), (ii) dehydroxylation of montmorillonite layers (570–710 °C) and (iii) reduction of Co bound in hardly reducible forms (above 710 °C). As previously, the spinel phase reduction onset temperature was determined, considering that reducibility may be a factor significantly determining the catalytic activity in the toluene combustion. The obtained results are shown in Figure 10b. For all materials, regardless of pH used during the adsorption of Co^2+^ ions, a very similar tendency is observed. The catalysts modified in the solution with the lowest Co(NO_3_)_2_ solution concentration (0.005 M) exhibit the strongest stabilization of the cobalt phase due to the relatively low Co content and high dispersion of the formed spinel phase. An increase in the concentration of the modifying solution to the range of 0.010 to 0.020 M allows the formation of Co_3_O_4_ nanoparticles with the lowest temperature of reduction beginning. On the other hand, a further increase in the Co(NO_3_)_2_ solution concentration leads to a slight deterioration of reducibility, which may be the result of too high an aggregation of spinel particles.

As in the case of the previously discussed preparations obtained by the modification in various volumes of the Co(NO_3_)_2_ solution, the surface composition analyses were also performed using XPS for the samples prepared by deposition at the different concentrations of the Co(NO_3_)_2_ solution. Table 4 displays the determined contents of individual elements, as well as a comparison of the bulk (XRF) and surface (XPS) compositions. With very similar trends in the content of Na and Co, it should be noted that the surface is definitely depleted in the deposited active phase of Co_3_O_4_.

**Figure 11:**
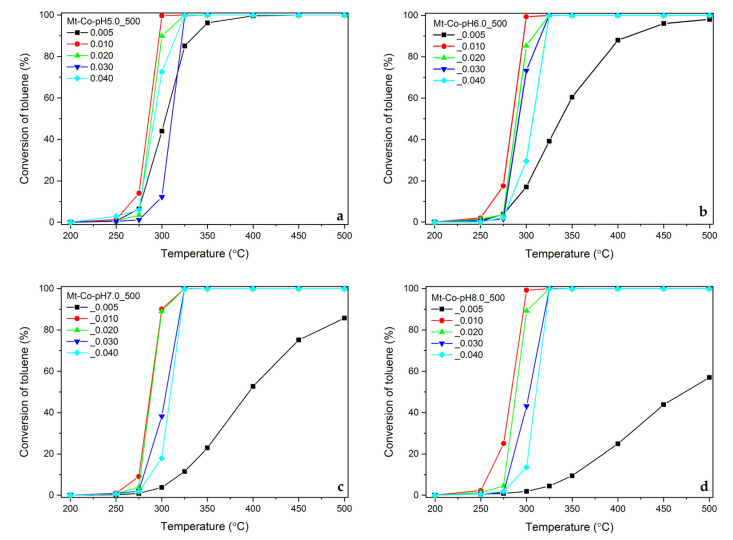
Influence of reaction temperature on toluene conversion over the calcined Mt-Co-pH5.0_500 (**a**), Mt-Co-pH6.0_500 (**b**), Mt-Co-pH7.0_500 (**c**) and Mt-Co-pH8.0_500 (**d**) samples obtained by deposition in various concentrations of Co(NO_3_)_2_ solution (500 mL) using NaOH as the pH-controlling agent.

Finally, the materials obtained by the modification in the Co(NO_3_)_2_ solution of various concentrations were tested as catalysts for the total oxidation of toluene (Figure 11).

**Figure 12:**
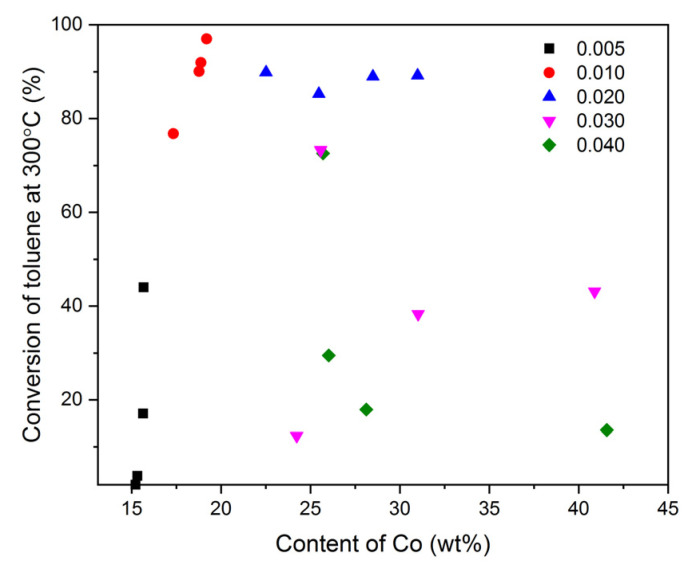
Influence of Co content on conversion of toluene achieved at 300 °C over the Mt-Co-pHx catalysts obtained by deposition in Co(NO_3_)_2_ solution (500 mL) of various concentrations using NaOH as the pH-controlling agent.

The aluminosilicate support with the deposited Co_3_O_4_ active phase provides a very high selectivity to the expected reaction products (CO_2_ and H_2_O). However, a significant influence of the concentration of the modifying solution on the activity of the spinel phase is observed. Definitely, the best results, regardless of pH used during the modification, were collected for the samples prepared in the Co(NO_3_)_2_ solutions with the concentrations of 0.010 and 0.020 M. Both lower and higher concentrations did not guarantee the appropriate content and dispersion of Co_3_O_4_ nanoparticles. An interesting overview of the discussed problem is found by the comparison of the toluene conversion achieved over the tested materials at 300 °C as a function of the Co content (Figure 12).

The greatest changes in the activity are observed in the case of the catalysts synthesized by adsorption in the Co(NO_3_)_2_ solutions with the concentrations of 0.005 and 0.010 M. With insignificant differences in the Co content, a clear increase in the toluene conversion is obtained at decreasing pH (the discussed effect is most noticeable in the case of the Mt-Co-pHx_500_0.005 catalysts). This most likely means that the deposition of Co^2+^ ions results in the formation of cobalt species interacting in different ways with the surface of the Mt support. Consequently, differences in reducibility and catalytic activity of the corresponding catalysts appear. As expected, the best catalytic results were found for the series of Mt-Co-pHx_500_0.010 and Mt-Co-pHx_500_0.020. It is worth noting, however, that even a significant increase in the Co content observed in the latter series did not cause any clear variations in the rate of the hydrocarbon substrate conversion. Against this background, the Mt-Co-pHx_500_0.030 and Mt-Co-pHx_500_0.040 materials are significantly worse, despite the high Co content.

The above discussion leads to the conclusion that too high a concentration of the modifying solution has a negative effect on the dispersion of the active phase. Relatively large particles of the Co_3_O_4_ phase demonstrate, on the one hand, poorer redox properties, and, on the other hand, are not sufficiently active in the studied catalytic process.

### 2.3. Effect of pH-Controlling Agent

The above-described donation of the Mt–PAA composite with Co^2+^ ions was carried out in the presence of NaOH, used as the pH-controlling agent. As shown by chemical analyses (bulk and surface), selected deposition conditions could favor the competitive adsorption of Na^+^ ions. Sodium admixtures remaining in the material after calcination could, in turn, affect the properties of the studied materials, including the catalytic performance. Therefore, it was decided to check to what extent the replacement of sodium hydroxide with another pH-controlling agent (KOH or NH_3_ solution) could change characteristics of the final samples. For the investigation, the modification at pH = 6.0 using 0.01 M Co(NO_3_)_2_ solution of the volume varying from 100 to 700 mL was selected.

**Figure 13:**
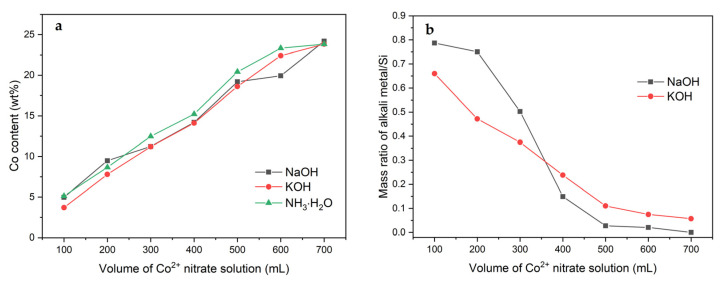
(**a**) Co content as well as (**b**) Na(K)/Si mass ratio in the calcined Mt–PAA composite modified in different volumes of 0.01 M Co(NO_3_)_2_ solution using various pH-controlling agents.

The kind of the pH-controlling agent has an insignificant influence on the Co content (Figure 13a). Regardless of whether NaOH is used during the modification, or is replaced by KOH or NH_3_ solution, the amount of Co incorporated changes in a very similar trend with increasing volume of the Co(NO_3_)_2_ solution. The content of alkaline admixtures after the calcination was also determined by XRF. In this respect, the aqueous NH_3_ solution has a definite advantage. Its use could lead to the possible appearance of NH_4_^+^ cations in the structure of the modified Mt–PAA composite, which during the thermal treatment decomposed into a proton form. The contents of Na and K in the catalysts obtained by the adsorption in various volumes of the Co(NO_3_)_2_ solution are compared in Figure 13b. The amount of alkali metal decreases with the increasing volume of the modifying solution. However, the adsorption of Na^+^ ions is more effective in the volume range up to 300 mL, whereas starting K^+^ cations are more easily incorporated.

**Figure 14:**
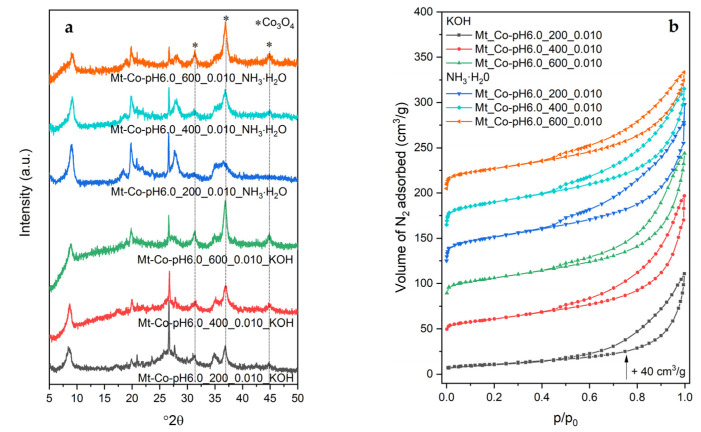
(**a**) XRD patterns and (**b**) N_2_ adsorption–desorption isotherms of the calcined Mt-Co-pH6.0 samples obtained by deposition in different volumes of 0.01 M Co(NO_3_)_2_ solution at pH = 6.0 using various pH-controlling agents.

The materials prepared using KOH or NH_3_ solution as the pH-adjusting agent were studied for structural properties. Selected diffraction patterns are presented in Figure 14a.

It should be noted that there are no significant differences in the phase composition of the tested materials, which, apart from the partially delaminated montmorillonite structure contain traces of mineral impurities (mainly quartz) and crystallites of the Co_3_O_4_ phase. In the case of the materials obtained in the presence of the aqueous NH_3_ solution, less ordering of the spinel structure is found (a significant broadening of the (311) diffraction peak) for the samples with the lower Co content. This effect is more interesting when considering the results of adsorption studies (Figure 14b and Table 5). The collected N_2_ adsorption isotherms and the determined textural parameters suggest that, using the aqueous solution of NH_3_, one can prepare the materials with a significant opening of the porous structure, even with small amounts of cobalt introduced. If we take into account the relatively high intensity of the (001) reflection observed in the diffractograms of the discussed samples, indicating the occurrence of the layered order, it should be presumed that in this case cobalt species are mainly formed in the spaces between the aluminosilicate particles. The effect of increasing porosity is possible due to a sufficiently large aggregation of Co_3_O_4_ particles.

The unlike behavior of the Mt–PAA system in the presence of the aqueous NH_3_ solution can be attributed to the appearance of another type of complexes. Co^2+^ aqua complexes formed in the NaOH and KOH solutions were most likely replaced by complexes with NH_3_, which consequently resulted in a change in the interaction of Co^2+^ complex ions with the surface of the Mt–PAA composite.

**Figure 15:**
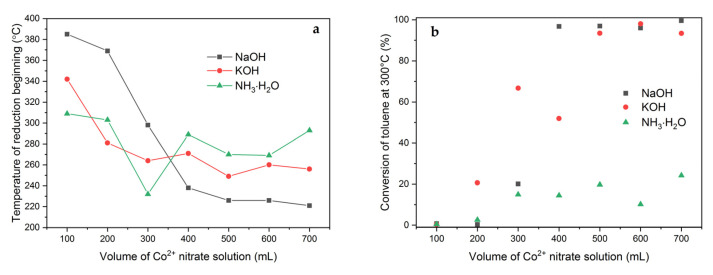
(**a**) Temperatures of reduction beginning determined from the H_2_-TPR measurements and (**b**) influence of volume of 0.01 M Co(NO_3_)_2_ solution and pH-controlling agent on conversion of toluene achieved at 300 °C.

The results of H_2_-TPR studies do not show clear differences in the mechanism of reduction of cobalt species, which for all tested samples occur in two basic forms—easily reducible Co_3_O_4_ nanoparticles and hardly reducible Co^2+^ cations firmly bonded to the aluminosilicate support. Taking into account the influence of reducibility of the spinel phase on the catalytic activity in the total oxidation of toluene, the dependence of the temperature at the beginning of its reduction on the volume of the Co(NO_3_)_2_ solution and the type of the pH-controlling agent used during the modification is analyzed in Figure 15a.

Regardless of the type of the pH-controlling agent used, along with the increase in the amount of Co introduced, the temperature at which the spinel phase reduction begins gradually decreases. This effect is most pronounced when NaOH is used and least visible for the aqueous NH_3_ solution. The presented dependencies clearly confirm that the most easily reducible Co_3_O_4_ nanoparticles are created for the Mt-Co-pH6.0 materials modified in the volume range of 400 to 700 mL of Co(NO_3_)_2_ solution in the presence of NaOH. Taking into account the very similar structure, textural parameters and Co content between the series of catalysts obtained using different pH-controlling agents, it can be assumed that the observed differences in reducibility are the result of different particle size distribution of the spinel phase and, possibly, their interaction with the clay surface. The worst in this context are the catalysts prepared using the aqueous NH_3_ solution, for which the increase in the Co content results, to the smallest extent, in a change in the temperature of the beginning of the Co_3_O_4_ phase reduction.

The Mt-Co-pH6.0 materials prepared with various pH-adjusting agents were finally tested as toluene combustion catalysts. For easier comparison of the activity of the tested materials, Figure 15b shows the values of the toluene conversion determined at 300 °C. It is evidently visible that the best catalytic results are observed in the presence of the Mt-Co-pH6.0 materials obtained by adsorption of Co^2+^ ions from 0.010 M Co(NO_3_)_2_ solution with volumes of 400–700 mL (in the case of pH control by adding NaOH) or 500–700 mL (pH control with KOH). Against this background, the catalysts synthesized with the use of the aqueous NH_3_ solution look very unfavorable.

## 3. Materials and Methods

### 3.1. Chemicals

Montmorillonite (natural Slovak bentonite, Jelšový potok), acrylic acid (98%, Acros Organics, France, extra pure), N,N′-methylenebisacrylamide (Sigma-Aldrich, St. Louis, MO, USA, 99%), ammonium persulfate (Chempur, Piekary Śląskie, Poland, pure p.a.), ammonia solution (28–30%, J. T. Baker, Philipsburg, NJ, USA), nitric acid (V) solution (65%, Sigma-Aldrich, Stainheim, Germany, puriss p.a.), cobalt(II) nitrate hexahydrate (Honeywell, Seelze, Ger-many, 98%), sodium hydroxide (Chempur, Piekary Śląskie, Poland, pure p.a.), potassium hydroxide (Lachner, Neratovice, Czech Republic, pure p.a.), toluene (Honeywell, Seelze, Germany, puriss p.a.) and nitrogen (Air Products, Świerzewo, Poland, grade 5.2).

### 3.2. Synthesis

Montmorillonite-poly(acrylic acid) (PAA–Mt) composite containing 50 wt% of the clay and 50 wt% of the hydrogel was prepared using the procedure described earlier in [47]. PAA–Mt was then modified by introducing various amounts of Co^2+^ cations by adsorption from an aqueous Co(NO_3_)_2_ solution. Then, 2 g of the obtained composite material was placed in a round-bottom flask of an appropriate volume (250, 500, 750 or 1000 mL), and then 100 to 700 mL of the Co(NO_3_)_2_ solution was added at the suitable concentration (0.005, 0.010, 0.020, 0.030 or 0.040 M). The Co^2+^ ion adsorption process was carried out for 24 h at a controlled temperature of 30 °C and pH (in the range of 5.0 to 8.0), while stirring the suspension using a magnetic stirrer at a speed of 500 rpm. Then, 0.1 M HNO_3_ and NaOH solutions were used to maintain a stable pH level (in selected cases the NaOH solution was replaced by a KOH or NH_3_ solution). After 24 h, which was sufficient to achieve adsorption equilibrium, the modified PAA–Mt composite was centrifuged and then dried in an oven at 65 °C for 48 h. In order to remove the polymer matrix and obtain a stable oxide form, calcination was carried out at 500 °C for 6 h at an air atmosphere, with a temperature increase of 1 °C/min. The obtained materials were denoted as Mt-Co-pH*x*_*v*_*c*, where *x* expresses the pH value kept during the modification, *v*—the volume of the Co(NO_3_)_2_ solution and *c*—its concentration.

### 3.3. Characterization

The crystalline structure of the materials was studied by X-ray powder diffraction (XRD). XRD patterns were collected on a Bruker D2 Phaser instrument using Cu Kα radiation (λ = 1.54184 Å) and a LYNXEYE detector within a 2θ range of 3 to 50° at a step of 0.02°.

Textural properties of the materials were determined by low-temperature adsorption of nitrogen at −196 °C using a Micromeritics ASAP 2020 sorptometer. The adsorption measurements were preceded by an outgassing procedure at 250 °C for 5 h under vacuum. Specific surface areas of the tested catalysts (S_BET_) were determined based on the Brunauer–Emmett–Teller (BET) model, while total pore volumes (V_total_) were calculated using a single-point adsorption value at relative pressure *p*/*p*_0_~0.99. The t-plot method was used to determine volume of micropores (V_micro_). Volume of mesopores (V_meso_) was obtained by applying the Barrett–Joyner–Halenda (BJH) model analyzing the desorption branches of the measured isotherms.

Chemical analysis of all samples was performed by X-ray fluorescence (XRF) using a Thermo Scientific ARL Quant’x spectrometer.

Temperature-programmed reduction measurements (H_2_-TPR) were carried out for the investigated samples (ca. 10 mg) using gaseous H_2_ as a reducing agent (a mixture containing 10 vol% of H_2_ in N_2_). A TA Instruments SDT Q600 thermoanalyzer was chosen as a detector for determination of reduction process progress. The H_2_-TPR runs were studied in a temperature range of 30 to 1000 °C, with a temperature increase of 20 °C/min, in the flow of the reducing mixture (flow rate through the measuring chamber was 60 mL/min).

X-ray photoelectron spectroscopy (XPS) measurements were conducted using a Prevac photoelectron spectrometer equipped with a monochromatized aluminum source AlKα (E = 1486.6 eV) and a hemispherical VG SCIENTA R3000 analyzer. To compensate for the charge on the surface of nonconductive samples, a low-energy electron flood gun (FS40A-PS) was used. The scale of the binding energy value was adjusted to a reference Si 2p peak at 103.0 eV. The Shirley background and fitting with the mixed function of Gauss and Lorentz (GL = 30) were used during interpretation of the spectra in the CasaXPS software.

### 3.4. Catalytic Activity

The total oxidation of toluene was chosen as a test reaction to recognize the catalytic activity of the developed Co-containing materials. An amount of 0.1 g of a sample (with a particle size kept within a range of 160 to 315 μm) was introduced into a quartz flow microreactor and placed in its central position on a quartz wool plug. Before a catalytic test, the sample was outgassed at 500 °C for 30 min in flowing air (100 mL/min) and then cooled down to 200 °C. When this temperature was reached, toluene dosing began by passing air through a saturator filled with liquid aromatic hydrocarbon to achieve the toluene vapor concentration of 1000 ppm in the reaction mixture. After 15 min, a first GC analysis of products was initialized. The conversion of toluene and yields of possible products were determined using a Bruker 450-GC gas chromatograph equipped with three columns (Molecular Sieve 5A for separation of O_2_, N_2_ and CO, Porapak S for separation of CH_4_, CO_2_ and H_2_O and Chromosorb WAW-DMCS for separation of aromatic compounds), a thermal conductivity detector and two flame ionization detectors, as well as a methanizer. The catalytic activity was studied at 200, 250, 275, 300, 325, 350, 400, 450 and 500 °C with 3 GC analyses (each analysis took 25 min) at a given temperature (heating rate between the temperature steps was 10 °C/min). The conversion results presented in the study were calculated as the average of three points obtained at a given temperature. Isothermal runs were also performed. In such cases, the reactor with the outgassed catalyst was cooled down to 290 °C, and after starting toluene dosing it was kept at this temperature for 24 h with the consecutive product analyses at intervals of 25 min.

## 4. Conclusions

In the presented work, the modification of the clay-hydrogel composite structure containing partially delaminated montmorillonite with incorporated water-swelling poly (acrylic acid) by adsorption of Co^2+^ ions from the aqueous Co(NO_3_)_2_ solution was thoroughly analyzed. Taking into account all the above-discussed contributions, it can be concluded that the conditions of Co^2+^ ions deposition (pH, volume and concentration of Co(NO_3_)_2_ solution, as well as a type of pH-controlling agent) play an important role in the course of active phase precursor adsorption, and a proper distribution of Co_3_O_4_ nanoparticles formed after calcination. The materials prepared under various conditions differ significantly in the Co content (determined mainly by the amount of the modifying solution used) and the dispersion/strength of interaction of the formed Co_3_O_4_ phase with the clay support (significant influence of the pH-controlling agent), which influence the observed catalytic properties. The properly selected modification conditions guarantee the synthesis of systems in which easily reducible Co_3_O_4_ nanoparticles disperse between exfoliated aluminosilicate packets, which protect them against sintering, creating a highly active, selective and stable mesoporous structure in the combustion of aromatic VOCs. For this reason, they can be seriously considered candidates for commercial application.

## Figures and Tables

**Table 1:** Textural parameters of the calcined Co_3_O_4_/Mt materials obtained by deposition in various volumes of 0.01 M Co(NO_3_)_2_ solution using NaOH as the pH-controlling agent.

Sample	Textural Parameters			
	S_BET_ (m^2^/g)	V_meso_ (cm^3^/g)	V_micro_ (cm^3^/g)	V_total_ (cm^3^/g)
Mt-Co-pH5.0_100	21	0.08	0.001	0.12
Mt-Co-pH5.0_200	32	0.13	0.001	0.17
Mt-Co-pH5.0_300	93	0.21	0.001	0.25
Mt-Co-pH5.0_400	55	0.17	0.001	0.18
Mt-Co-pH5.0_500	86	0.18	0.003	0.22
Mt-Co-pH5.0_600	32	0.11	0.003	0.15
Mt-Co-pH5.0_700	93	0.19	0.003	0.22
Mt-Co-pH6.0_100	14	0.06	0.001	0.09
Mt-Co-pH6.0_200	17	0.10	0.001	0.11
Mt-Co-pH6.0_300	27	0.13	0.001	0.15
Mt-Co-pH6.0_400	63	0.19	0.001	0.20
Mt-Co-pH6.0_500	94	0.20	0.005	0.23
Mt-Co-pH6.0_600	96	0.21	0.001	0.26
Mt-Co-pH6.0_700	94	0.21	0.005	0.25
Mt-Co-pH7.0_100	15	0.03	0.001	0.06
Mt-Co-pH7.0_200	20	0.08	0.001	0.12
Mt-Co-pH7.0_300	31	0.13	0.001	0.18
Mt-Co-pH7.0_400	23	0.13	0.001	0.15
Mt-Co-pH7.0_500	55	0.19	0.001	0.23
Mt-Co-pH7.0_600	82	0.19	0.009	0.23
Mt-Co-pH7.0_700	92	0.23	0.006	0.27
Mt-Co-pH8.0_100	20	0.06	0.003	0.09
Mt-Co-pH8.0_200	26	0.10	0.001	0.15
Mt-Co-pH8.0_300	31	0.11	0.001	0.13
Mt-Co-pH8.0_400	40	0.13	0.001	0.16
Mt-Co-pH8.0_500	78	0.20	0.002	0.21
Mt-Co-pH8.0_600	96	0.29	0.004	0.30
Mt-Co-pH8.0_700	103	0.26	0.004	0.30

**Table 2:** Surface composition of the calcined Co_3_O_4_/Mt materials obtained by deposition in various volumes of 0.01 M Co(NO_3_)_2_ solution using NaOH as the pH-controlling agent.

Sample	Content (% at.)						Molar Ratio		
	Si	Al	O^2−^	OH^−^	Na	Co	Co^2+^/Co^3+^	Co/Si Bulk ^a^	Co/Si Surface ^b^
**Mt-Co-pH5.0**									
100	17.61	5.83	2.71	52.01	16.26	5.58	1.5	0.28	0.32
300	20.93	6.97	7.14	48.64	10.74	5.59	1.2	0.53	0.27
500	22.68	7.70	2.34	59.16	2.76	5.34	1.7	0.69	0.24
700	22.38	7.15	3.02	58.72	-	8.74	1.7	0.79	0.39
**Mt-Co-pH6.0**									
100	17.66	5.79	0.70	55.06	18.20	2.59	1.4	0.23	0.15
300	18.61	5.98	1.31	53.95	12.88	7.28	2.3	0.50	0.39
500	21.58	6.76	7.17	54.08	-	10.42	1.5	0.81	0.48
700	21.47	6.61	6.03	54.29	-	11.59	1.4	1.11	0.54
**Mt-Co-pH7.0**									
100	14.43	4.04	1.50	53.74	22.93	3.36	1.2	0.31	0.23
300	18.88	5.71	5.59	48.97	12.81	8.04	1.8	0.47	0.43
500	19.65	5.01	13.02	42.87	6.56	12.90	1.1	0.84	0.66
700	21.22	6.27	9.43	50.72	-	12.38	1.1	1.29	0.58
**Mt-Co-pH8.0**									
100	14.49	4.48	0.69	50.68	26.77	2.88	1.9	0.22	0.20
300	18.66	5.52	13.65	39.35	13.95	8.87	1.0	0.45	0.48
500	19.22	5.20	12.06	43.50	5.84	14.18	1.7	0.83	0.74
700	20.43	6.16	8.81	49.17	2.77	12.68	1.0	1.48	0.62

^a^ determined by XRF; ^b^ determined by XPS.

**Table 3:** Textural parameters of the calcined Co_3_O_4_/Mt materials obtained by deposition in various concentrations of Co(NO_3_)_2_ solution (500 mL) using NaOH as the pH-controlling agent.

Sample	Textural Parameters			
	S_BET_ (m^2^/g)	V_meso_ (cm^3^/g)	V_micro_ (cm^3^/g)	V_total_ (cm^3^/g)
Mt-Co-pH5.0_500_0.005	37	0.11	0.002	0.15
Mt-Co-pH5.0_500_0.010	86	0.18	0.003	0.22
Mt-Co-pH5.0_500_0.020	100	0.19	0.005	0.27
Mt-Co-pH5.0_500_0.030	101	0.19	0.005	0.28
Mt-Co-pH5.0_500_0.040	85	0.19	0.004	0.22
Mt-Co-pH6.0_500_0.005	28	0.11	0.001	0.17
Mt-Co-pH6.0_500_0.010	94	0.20	0.005	0.23
Mt-Co-pH6.0_500_0.020	93	0.18	0.005	0.26
Mt-Co-pH6.0_500_0.030	100	0.21	0.005	0.30
Mt-Co-pH6.0_500_0.040	90	0.16	0.005	0.18
Mt-Co-pH7.0_500_0.005	24	0.09	0.001	0.15
Mt-Co-pH7.0_500_0.010	55	0.19	0.001	0.23
Mt-Co-pH7.0_500_0.020	94	0.19	0.004	0.25
Mt-Co-pH7.0_500_0.030	107	0.21	0.007	0.29
Mt-Co-pH7.0_500_0.040	90	0.20	0.001	0.22
Mt-Co-pH8.0_500_0.005	20	0.08	0.001	0.14
Mt-Co-pH8.0_500_0.010	78	0.20	0.002	0.21
Mt-Co-pH8.0_500_0.020	100	0.26	0.005	0.26
Mt-Co-pH8.0_500_0.030	110	0.19	0.007	0.27
Mt-Co-pH8.0_500_0.040	105	0.20	0.008	0.24

**Table 4:** Surface composition of the calcined Co_3_O_4_/Mt materials obtained by deposition in various concentrations of Co(NO_3_)_2_ solution (500 mL) using NaOH as the pH-controlling agent.

Sample	Content (% at.)						Molar Ratio		
	Si	Al	O^2−^	OH^−^	Na	Co	Co^2+^/Co^3+^	Co/Si Bulk ^a^	Co/Si Surface ^b^
**Mt-Co-pH5.0_500**									
0.005	21.23	6.78	2.34	59.16	9.51	4.88	1.5	0.65	0.23
0.010	22.68	7.70	4.24	57.21	2.76	5.34	1.7	0.69	0.24
0.020	20.40	5.52	9.89	47.60	-	16.58	1.2	1.00	0.81
0.030	20.07	5.27	7.57	49.84	-	17.24	1.8	1.12	0.86
0.040	21.92	6.67	6.45	54.50	-	10.47	1.7	1.22	0.48
**Mt-Co-pH6.0_500**									
0.005	18.68	5.86	5.84	48.62	16.04	4.95	1.7	0.68	0.26
0.010	21.58	6.76	7.17	54.08	-	10.42	1.5	0.81	0.48
0.020	20.74	5.93	8.11	50.11	-	15.11	1.2	1.21	0.73
0.030	22.44	6.70	6.54	54.88	-	9.51	1.9	1.21	0.42
0.040	20.55	5.74	9.62	49.78	-	14.29	1.4	1.25	0.70
**Mt-Co-pH7.0_500**									
0.005	17.55	5.18	4.28	49.32	18.27	5.40	2.3	0.70	0.31
0.010	19.65	5.01	13.02	42.87	6.56	12.90	1.1	0.84	0.66
0.020	22.53	7.18	9.74	51.55	-	9.07	1.1	1.45	0.40
0.030	19.88	5.29	9.03	48.76	-	17.05	1.3	1.65	0.86
0.040	19.46	5.46	8.24	49.52	-	17.34	1.3	1.40	0.89
**Mt-Co-pH8.0_500**									
0.005	16.48	4.56	4.97	47.74	21.87	4.40	1.5	0.72	0.27
0.010	19.22	5.20	12.06	43.50	5.84	14.18	1.7	0.83	0.74
0.020	20.58	5.33	8.10	48.84	-	17.16	1.7	1.69	0.83
0.030	18.94	3.20	14.13	39.77	-	23.95	1.9	2.83	1.26
0.040	18.93	3.80	11.42	44.94	-	20.90	1.9	3.01	1.10

^a^ determined by XRF; ^b^ determined by XPS.

**Table 5:** Textural parameters of the calcined Co_3_O_4_/Mt materials obtained by deposition in different volumes of 0.01 M Co(NO_3_)_2_ solution at pH = 6.0 using KOH and NH_3_ solution as the pH-controlling agents.

Sample	Textural Parameters			
	S_BET_ (m^2^/g)	V_meso_ (cm^3^/g)	V_micro_ (cm^3^/g)	V_total_ (cm^3^/g)
KOH				
Mt-Co-pH6.0_100_0.010	27	0.08	0.001	0.09
Mt-Co-pH6.0_200_0.010	39	0.11	0.002	0.11
Mt-Co-pH6.0_300_0.010	51	0.14	0.001	0.14
Mt-Co-pH6.0_400_0.010	75	0.16	0.001	0.17
Mt-Co-pH6.0_500_0.010	91	0.15	0.005	0.18
Mt-Co-pH6.0_600_0.010	94	0.17	0.005	0.19
Mt-Co-pH6.0_700_0.010	98	0.21	0.005	0.21
**NH_3_·H_2_O**				
Mt-Co-pH6.0_100_0.010	93	0.10	0.007	0.13
Mt-Co-pH6.0_200_0.010	113	0.15	0.006	0.19
Mt-Co-pH6.0_300_0.010	121	0.20	0.005	0.21
Mt-Co-pH6.0_400_0.010	108	0.16	0.005	0.19
Mt-Co-pH6.0_500_0.010	101	0.16	0.004	0.19
Mt-Co-pH6.0_600_0.010	97	0.15	0.004	0.17
Mt-Co-pH6.0_700_0.010	107	0.18	0.005	0.21

## Data Availability

The data presented in this study are available on request from the corresponding author.

## Sample Availability

Samples of the compounds are not available from the authors.
